# The relationship between workplace bullying and adaptive performance in junior nurses: the mediating role of emotion regulation and work engagement

**DOI:** 10.3389/fpubh.2026.1805252

**Published:** 2026-03-25

**Authors:** Fulan Du, Min Tan, Lanjun Luo, Siyuan Li, Xuemei Wei

**Affiliations:** 1 Affiliated Hospital of North Sichuan Medical College, Nanchong, Sichuan, China; 2 School of Management, North Sichuan Medical College, Nanchong, Sichuan, China; 3 Sichuan Mianyang 404 Hospital, Mianyang, Sichuan, China

**Keywords:** emotional regulation, job performance, nurses, occupational stress, work engagement, workplace bullying

## Abstract

**Background:**

Amidst the rapidly evolving global healthcare environment, junior nurses must develop greater adaptability. However, issues such as workplace bullying significantly undermine their adaptive performance. Guided by the Job Demands-Resources Theory and Emotion Regulation Theory, this study examines the chain-mediating role of cognitive reappraisal, expressive suppression, and work engagement in the relationship between workplace bullying and adaptive performance.

**Methods:**

From April to May 2024, a cross-sectional survey was conducted in 17 hospitals in Southwest China. The study utilized a comprehensive set of measurement tools, including the General Information Questionnaire, the Negative Acts Questionnaire-Revised, the Emotion Regulation Questionnaire, the Work Engagement Questionnaire, and the Adaptive Performance Questionnaire. All statistical analyses were performed with SPSS and Mplus.

**Results:**

A total of 988 junior nurses were included in this study. Adaptive performance was negatively correlated with workplace bullying (*r* = −0.335) and expressive suppression (*r* = −0.180) and positively correlated with cognitive reappraisal (*r* = 0.556) and work engagement (*r* = 0.576) (all *p* < 0.01). The indirect effect of workplace bullying on adaptive performance via emotion regulation and work engagement was significant (standardized indirect effect = −0.188, 95% BC CI [−0.238, −0.138]), accounting for 51.8% of the total standardized effect.

**Conclusion:**

There was a significant negative correlation between workplace bullying and adaptive performance of junior nurses, in which cognitive reappraisal, expressive suppression, and work engagement played a mediating role. This suggests that when healthcare institutions and nursing administrators develop strategies to enhance adaptive performance, they should mitigate workplace bullying and enhance cognitive reappraisal capabilities, reduce expressive suppression tendencies, and strengthen work engagement levels. However, the cross-sectional design precludes causal inferences, and the regional sample may limit generalizability.

## 1 Introduction

In the increasingly complex modern nursing practice environment, the ability of nurses to flexibly respond to evolving work tasks and patient needs has become a critical determinant of organizational sustainability (1, 2). As the new generation of nursing staff and a vital future talent pool, Junior nurses (with ≤5 years of clinical experience (3)) play a crucial role in responding to public health emergencies and addressing the complex care needs of an aging society. Their adaptive performance levels directly impact clinical responsiveness and patient safety outcomes (4, 5).

Unlike most professions, the core of nursing practice is characterized by high frequency, high intensity, and unpredictability. This inherent demand for “adaptive performance,” with its immediacy and direct impact on patient safety, constitutes a vital competency in the nursing profession (5). Junior nurses are in a critical transition period from students to independent practitioners. They face numerous challenges, including role transition, consolidating their knowledge base, and establishing interpersonal relationships, all while maintaining composure and professionalism in a high-pressure, uncertain environment (6–8). This “newcomer” status places them at the forefront of occupational stress, while their psychological resources and coping mechanisms are still developing. Therefore, investigating the factors influencing their adaptive performance has unique preventive value.

Some nurses with weaker adaptive performance struggle to quickly adapt to the rapid changes and diverse tasks in clinical environments (5). This increases their psychological burden and leads to poor work performance. It may also trigger talent loss, thereby raising the costs of nursing human resource management and posing a threat to the stability of global healthcare systems (6, 9, 10). Therefore, enhancing the adaptive performance of low-seniority nurses and strengthening their core adaptive capabilities is essential for personal growth, industry development, and achieving global health goals.

Adaptive performance refers to behaviors voluntarily undertaken by employees to adapt to new environments, work situations, or unforeseen events (11). It effectively compensates for the limitations of traditional static task performance in dynamic contexts (12). For junior nurses working in complex, ever-changing clinical environments, its core dimensions focus on “stress and emergency response” and “interpersonal and cultural adaptation”: the former ensures rapid response and patient safety in emergencies, while the latter enhances nursing efficiency through harmonious nurse–patient relationships and team collaboration (13). Studies have confirmed that nurses with high adaptive performance can more effectively cope with unexpected situations and reduce errors, whereas low adaptive performance leads to inefficiency and compromised nursing quality (5, 12). Therefore, an in-depth exploration of the factors influencing adaptive performance among junior nurses is a central issue for optimizing nursing human resource management and improving organizational performance.

It is worth noting that junior nurses are a high-risk group for workplace bullying, with an incidence rate as high as 57.1% (14). Workplace bullying (WPB) refers to individuals repeatedly experiencing negative behaviors such as harassment, exclusion, or organizational injustice in the workplace over a long period (15). As a severe occupational stressor, workplace bullying has proven to impair employee performance through various pathways. Mehmood et al. (16–18) found that workplace bullying undermines employees’ psychological well-being, which, in turn, affects their performance. Omotoye et al. (19) further indicated that workplace bullying and incivility significantly reduce employees’ job satisfaction and work engagement, thereby diminishing their adaptive performance. Specifically, workplace bullying imposes substantial psychological distress and emotional burden on nurses (20), impairing their cognitive flexibility and emotional control. This makes them more prone to decision hesitation, emotional dysregulation, and operational errors in emergencies (21, 22). At the same time, workplace bullying undermines trust relationships among junior nurses and their colleagues or supervisors, hindering their interpersonal adaptation and cultural integration (23). In summary, workplace bullying plays a key role among the various elements influencing adaptive performance. A deeper exploration of its impact mechanism on adaptive performance is essential for better understanding and addressing this phenomenon. Based on this, the present study proposes Hypothesis 1: Workplace bullying can directly influence adaptive performance.

Emotion regulation theory provides a micro-mechanism perspective for understanding how individuals cope with workplace bullying. This theory suggests that when individuals encounter adverse events such as workplace bullying, they employ emotion regulation strategies to maintain a balance in their physical and mental health. Among these strategies, cognitive reappraisal and expressive suppression are the two most fundamental (24). Cognitive reappraisal involves adjusting one’s emotional experience by changing the way one interprets an event. This approach helps individuals positively reframe workplace challenges, alleviating negative emotions and stress (25), and thus enabling them to devote more psychological resources to enhancing adaptive performance. Expressive suppression, on the other hand, regulates emotions by inhibiting emotional expression behaviors. However, frequent use of this strategy consumes psychological resources and is associated with increased negative emotions and cognitive load (26), which may hinder the development of adaptive performance.

The Job Demands-Resources (JD-R) theory (27) provides a comprehensive macro-theoretical framework for understanding how emotion regulation strategies mediate the relationship between job demands and work outcomes. According to the JD-R theory, workplace bullying is considered a high-intensity negative job demand. In response to this demand, the emotion regulation strategies individuals employ constitute necessary personal resources: cognitive reappraisal, as an adaptive strategy, helps nurses effectively manage their emotions, accumulate and maintain cognitive, affective, and social resources, and activate a gain pathway, which in turn enhances work engagement (28, 29). Conversely, expressive suppression, as a maladaptive strategy, consumes limited resources and activates a loss pathway, potentially leading to decreased work engagement (28). Within this framework, work engagement serves as a crucial transmission mechanism through which emotion regulation strategies influence adaptive performance. Characterized by positive emotions and high cognitive flexibility, a state of high work engagement has been consistently shown by multiple studies to predict adaptive performance positively (11, 30). Based on this, the present study proposes:

Hypothesis 2a: Cognitive reappraisal mediates the relationship between workplace bullying and adaptive performance among junior nurses.

Hypothesis 2b: Expressive suppression mediates the relationship between workplace bullying and adaptive performance among junior nurses.

Hypothesis 2c: Work engagement mediates the relationship between workplace bullying and adaptive performance among junior nurses.

Hypothesis 3: Cognitive reappraisal and expressive suppression sequentially influence work engagement, forming a chain mediation pathway between workplace bullying and adaptive performance in junior nurses.

Although the importance of Emotion regulation and work engagement for adaptive performance is recognized, significant gaps exist in current research: First, there is relatively insufficient investigation into the predictors of adaptive performance, particularly the mechanisms through which negative experiences (e.g., bullying) operate, specifically for junior nurses, a group particularly susceptible to environmental influences. Second, the complex relationships among workplace bullying, Emotion regulation strategies (cognitive reappraisal, expressive suppression), work engagement, and adaptive performance, especially whether and how these two Emotion regulation strategies form a chain mediation pathway via work engagement linking bullying to adaptive performance, have not been thoroughly examined within the junior nurse population. Given the urgency of fostering adaptive performance in this group, a deeper understanding of these mechanisms is crucial.

Therefore, grounded in the JD-R and Emotion regulation Theory, this study aims to construct and test a chain mediation model. It examines how workplace bullying affects junior nurses’ adaptive performance through the multiple mediating roles of cognitive reappraisal, expressive suppression, and work engagement. The findings are intended to provide a reference for developing targeted interventions to enhance adaptive performance levels among junior nurses.

## 2 Methods

### 2.1 Study design

This study used a cross-sectional design and mediation analysis, which was implemented with the STROBE checklist.

### 2.2 Participants

This study is a cross-sectional study conducted from April to May 2024, using convenience sampling to administer an online questionnaire survey via Questionnaire Star to junior nurses in hospitals of all levels in Sichuan Province, China. A convenience sampling strategy was adopted, consistent with the exploratory nature of this research, which aims to examine preliminary relationships among variables within a specific, hard-to-reach population. Given the logistical challenges and resource constraints of constructing a comprehensive sampling frame for all junior nurses nationwide, convenience sampling offered a practical and efficient approach to data collection. To enhance sample diversity and partially mitigate inherent selection bias, we recruited participants from 17 hospitals spanning four distinct geographical regions of Sichuan Province: Western Sichuan (*n* = 2), Northeastern Sichuan (*n* = 5), Central Sichuan (*n* = 7), and Southern Sichuan (*n* = 3). These hospitals included a mix of tertiary and secondary institutions located in both urban and suburban areas. While this method provided a valuable opportunity to gather data from a substantial sample, the non-probability sampling means that the findings may not be fully generalizable to other geographical or healthcare contexts. Therefore, the study interprets external validity with caution and views these results as providing foundational insights for future, more representative research. The target population comprised registered junior nurses with ≤5 years of clinical experience currently engaged in frontline work.

All participants met the following criteria: (a) Registered practicing nurses, (b) Currently engaged in clinical frontline nursing work with at least 6 months of clinical nursing experience and ≤5 years of work experience (excluding internship experience), (c) Agreed to participate in this study and signed an online informed consent form. Exclusion criteria: (a) Visiting trainees, (b) Experienced stressful events such as divorce and death of relatives in the last 6 months (31). (c) Received psychiatric medication or psychological therapy within the past 6 months.

The sample size was calculated using the formula for cross-sectional studies: *N* = (μ_1-*α*/2_ *σ*/*δ*)^2^, with a significance level of *α* = 0.05 and *μ_1-α/2_* = 1.96. *σ* represents the standard deviation of the adaptive performance scale score, and δ represents the allowable error. Based on the results of a 60-participant pre-survey, the standard deviation of adaptive performance scores among junior nurses was *σ* = 0.60. The allowable error *δ* was set as 8% of the standard deviation. The calculated sample size was approximately 600. Given a 20% invalid questionnaire rate, the final target sample size was set at least 750 participants.

### 2.3 Data collection

This study uses the “Questionnaire Star” platform to design electronic informed consent forms and formal questionnaires and send them to participants through generated links. This study obtained research permission from the head of the nursing department in each research environment, and the researchers distributed online survey links to the study participants with the assistance and support of nurse managers and nurses. Before answering the questions, the participants were introduced to the purpose, significance, inclusion and exclusion criteria, and precautions of the survey. Each participant completed the electronic informed consent before entering the formal questionnaire. This study follows the principles of informed consent, voluntariness, and anonymity. To avoid missing values, all items in the questionnaire are set as compulsory questions. An ID can only submit a questionnaire once. To encourage participants to answer carefully, they can participate in the lottery and receive 1–5 RMB after submitting the questionnaire. At the same time, to ensure the quality of the questionnaire, all the questionnaires were reviewed by the researchers in the background, and the money was distributed after the standard was met. In the investigation process, close contact with nurse managers and nurses, dynamic monitoring of filling, and timely communication to address the problem.

A total of 1,082 online questionnaires were collected. After excluding responses with completion times under 5 min and those exhibiting repetitive patterned responses, 988 valid questionnaires were retained for analysis.

### 2.4 Variables and measurements

#### 2.4.1 General information questionnaire

The researchers compiled the questionnaire, including gender, marital status, highest level of nursing education, hospital grade, years of experience, average work hours per day, average monthly income, and occupational exposure history.

#### 2.4.2 The negative acts questionnaire-revised (NAQ-R)

The scale is a 22-item scale compiled by Einarsen et al. (32) and revised by Xun et al. (33). It includes three dimensions: work-related bullying (9 items), person-related bullying (9 items), and organizational injustice (4 items). The scale is scored on a Likert-5 scale with the responses relating to the frequency of the experiences within the last 6 months as 1 (=never), 2 (=now and then), 3 (=monthly), 4 (=weekly), and 5 (=daily). The total score on the scale ranged from 22 to 110, with a higher score indicating more severe workplace bullying. The Cronbach’s alpha of the questionnaire in this study was 0.965.

#### 2.4.3 Emotion regulation questionnaire (ERQ)

The ERQ is a 10-item scale created by Gross and John (34) to assess individuals’ ability to regulate their emotions. It includes two dimensions: cognitive reappraisal (6 items) and expressive suppression (4 items). The scale is scored on a Likert-7 scale, ranging from “strongly disagree” to “strongly agree.” A higher score indicates that the individual is more adept at using Emotion regulation strategies. The Cronbach’s alpha of the two subscales was 0.963 for cognitive reappraisal and 0.918 for expressive suppression.

#### 2.4.4 Work engagement questionnaire (WEQ)

The scale is a 3-item scale created by Schaufeli et al. (35) to measure work engagement. It includes three items: “At my work, I feel bursting with energy,” “I am enthusiastic about my job,” and “I am immersed in my work.” The scale is scored on a Likert-5 scale, ranging from “strongly disagree” to “strongly agree.” The total score on this scale ranges from 3 to 15, with higher scores indicating a higher level of work engagement. The Cronbach’s alpha of the questionnaire in this study was 0.912.

#### 2.4.5 Adaptive performance questionnaire (WPQ)

The scale is compiled by Tao (13) to measure employees’ abilities to adjust their behaviors to satisfy work demands. The original scale contains 25 items measuring four domains of adaptive performance: handling emergencies and unpredictable situations (7 items), solving problems creatively (4 items), learning (6 items), and demonstrating culture and interpersonal adaptability (8 items). To compose a concise scale with the most relevant items for our study, we selected two subscales: handling emergencies and unpredictable situations, and demonstrating culture and interpersonal adaptability, 15 items in total. The scale is scored on a Likert-5 scale, ranging from “strongly disagree” to “strongly agree.” The total score on this scale ranges from 15 to 75, with higher scores indicating a higher level of adaptive performance. The Cronbach’s alpha of the questionnaire in this study was 0.972. This study used confirmatory factor analysis to examine the construct validity of the shortened Adaptive Performance Questionnaire. The model fit indices were as follows: *χ*^2^ = 529.529, df = 82, RMSEA = 0.074, CFI = 0.975, TLI = 0.968, SRMR = 0.028. All fit indices met acceptable standards, indicating that the scale possesses good construct validity.

The Cronbach’s *α* values for the NAQ-R (0.965), cognitive reappraisal (0.963), and adaptive performance (0.972) exceeded 0.95. While these values indicate high internal consistency, they may also suggest potential item redundancy. Given that all items were retained from validated instruments and demonstrated acceptable content validity, we considered the scales acceptable for use. The full versions of all instruments used in this study are provided in Supplementary material.

### 2.5 Statistical analysis

In this study, we used IBM SPSS Statistics 26.0 and MPLUS 8.3 for data analysis and structural equation modeling. The demographics and variables were described by mean and standard deviation, frequency, and percentage. Independent t-tests and one-way analysis of variance (ANOVA) were utilized to compare demographic characteristics influencing adaptive performance among junior nurses. To explore these relationships, *Pearson* correlation analysis was conducted on the normally distributed continuous variables, including workplace bullying, cognitive reappraisal, expressive suppression, work engagement, and adaptive performance. Then, the study built the SEM using MPLUS 8.3. The model fit indices included the comparative fit index (CFI), the Tucker-Lewis index (TLI), and the root-mean-square error of approximation (RMSEA) (36). A value of CFI and TLI exceeding 0.90 and an RMSEA value between 0 and 0.08 generally represent the model’s acceptable goodness of fit (36). Additionally, mediating effects were tested using the bootstrap method with 5,000 resamples, where effects were considered statistically significant if the 95% confidence interval (CI) did not include 0.

### 2.6 Ethical considerations

This study was approved by the Medical Ethics Committee of North Sichuan Medical College (2024ER112-1) and complied with the Declaration of Helsinki. All participants signed an online informed consent form before participating in the study. Moreover, we kept the data of all participants confidential.

## 3 Results

### 3.1 Common method bias test

This study employed both Harman’s single-factor test and the unmeasured latent method construct (ULMC) technique to test for common method bias. First, Harman’s single-factor test was conducted to examine the presence of common method bias (37). The results showed that eight factors with eigenvalues greater than 1 were extracted, and the largest factor explained 36.804% of the total variance, which is below the recommended threshold of 40%. Thus, common method bias was not a significant concern in this study.

Second, the ULMC approach was used to further test for common method bias. A common method factor was added to the baseline model, and the improvement in model fit was examined. The inclusion of the common method factor resulted in a significant chi-square change (Δ*χ^2^* = 521.273, Δ*df* = 1). However, the chi-square difference is sensitive to sample size, so other fit indices were also considered. The results showed that after adding the common method factor, the CFI and TLI increased by 0.027 and 0.029, respectively, and the RMSEA decreased by 0.006. All these changes were within 0.05, indicating no substantial improvement in model fit. Therefore, common method bias was not a serious issue in this study (38).

### 3.2 Demographic characteristic

A total of 988 junior nurses were included in this study. The majority were female (90.4%), unmarried (75.3%), and held a junior college diploma or lower qualification (55.6%). Most worked in tertiary hospitals (89.0%), had 1–3 years of clinical experience (42.1%), worked over 8 h daily (61.6%), and earned an average monthly income of 3,000–6,000 RMB (62.0%). Additionally, 38.2% of junior nurses had experienced occupational exposure (Table 1).

**Table 1:** Description and univariate analysis of adaptive performance among junior nurses (*N* = 988).

Variable	*N* (%)	Adaptive performance M (SD)	*t/F*	*P*
Gender			0.741^a^	0.460
Male	95 (9.6)	60.11 (14.34)		
Female	893 (90.4)	58.98 (10.97)		
Marital status			−2.087^a^	0.037
Unmarried	744 (75.3)	58.66 (11.31)		
Married	244 (24.7)	60.40 (11.30)		
Highest level of nursing education			5.709^b^	0.003
Associate degree or less	550 (55.6)	60.10 (11.50)		
Bachelor’s degree	428 (43.3)	57.72 (11.01)		
Master’s degree or above	10 (1.1)	62.20 (10.04)		
Hospital grade			−0.252^a^	0.801
Grade III	879 (89.0)	59.06 (11.18)		
Grade II	109 (11.0)	59.35 (12.52)		
Years of experience			5.098^b^	0.006
6 months to 1 year	226 (22.9)	57.31 (11.62)		
1 year to 3 years	416 (42.1)	58.98 (11.15)		
3 years to 5 years	346 (35.0)	60.38 (11.22)		
Average work hours per day			3.067^a^	0.002
≤8 h	379 (38.4)	60.49 (10.82)		
>8 h	609 (61.6)	58.22 (11.56)		
Average monthly income (RMB)			<0.001^b^	1.000
<3,000	227 (23.0)	59.09 (12.10)		
3,000–6,000	613 (62.0)	59.09 (11.29)		
>6,000	148 (15.0)	59.07 (10.29)		
Occupational exposure history			−4.846^a^	<0.001
Yes	377 (38.2)	56.89 (10.95)		
No	611 (61.8)	60.45 (11.36)		

^a^*t*-value for independent samples *t*-test; ^b^*F*-value for one-way ANOVA; M, mean; SD, standard deviation.

The univariate analysis revealed that adaptive performance scores differed significantly across marital status, education level, years of experience, daily working hours, and history of occupational exposure (*p* < 0.05).

### 3.3 Descriptive statistics and *Pearson* correlation analysis

The average adaptive performance score for junior nurses was 59.09 ± 11.33. Their scores for cognitive reappraisal, expressive suppression, and work engagement were 31.72 ± 7.81, 14.30 ± 6.68, and 11.26 ± 2.76, respectively (Table 2).

**Table 2:** Descriptive statistics and correlation analysis of each variable (*N* = 988).

Constructs	M (SD)	1	2	3	4	5
1 WPB	27.39 (10.38)	1				
2 Cognitive reappraisal	31.72 (7.81)	−0.207^**^	1			
3 Expression suppression	14.30 (6.68)	0.139^**^	−0.096^**^	1		
4 Work engagement	11.26 (2.76)	−0.283^**^	0.528^**^	−0.178^**^	1	
5 Adaptive performance	59.09 (11.33)	−0.335^**^	0.556^**^	−0.180^**^	0.576^**^	1

^**^p < 0.01.

This study used the *Pearson* test to test the correlation between variables. Workplace bullying was significantly negatively correlated with cognitive reappraisal (*r* = −0.207, *p* < 0.01), work engagement (*r* = −0.283, *p* < 0.01), and adaptive performance (*r* = −0.335, *p* < 0.01) and significantly positively correlated with --expressive suppression (*r* = 0.139, *p* < 0.01). Cognitive reappraisal was significantly positively correlated with work engagement (*r* = 0.528, *p* < 0.01) and adaptive performance (*r* = 0.556, *p* < 0.01), and expressive suppression was significantly negatively correlated with work engagement (*r* = −0.178, *p* < 0.01) and adaptive performance (*r* = −0.180, *p* < 0.01) (Table 2).

### 3.4 Testing the research hypothesis and analyzing the parameter estimates

**Figure 1:**
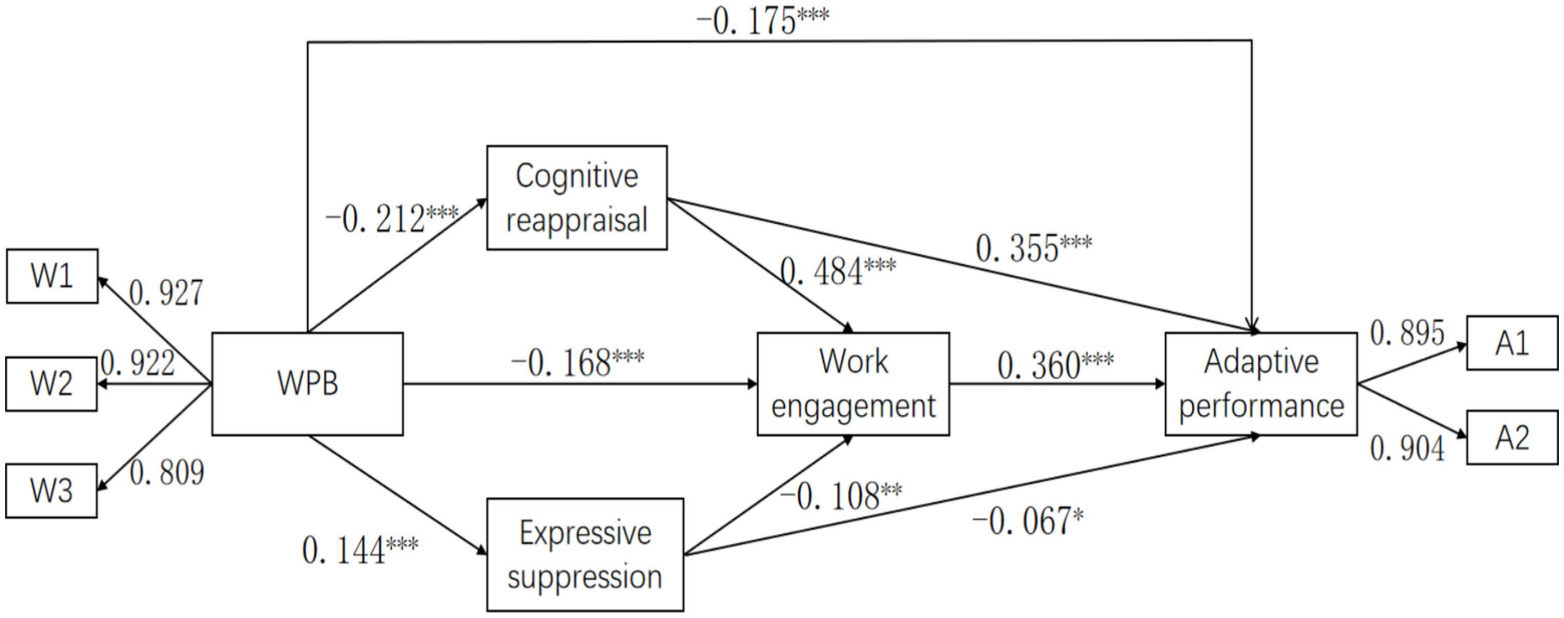
The details of the standardized coefficients. W1 = Work-related bullying; W2 = Person-related bullying; W3 = Organizational injustice; A1 = Handling emergencies and unpredictable situations; A2 = Demonstrating culture and interpersonal adaptability. ^*^p < 0.05, ^**^p < 0.01, ^***^p < 0.001.

This study used Mplus 8.3 to construct a structural equation model. In the model specification, the study included workplace bullying as an exogenous latent variable, with its three dimensions (work-related bullying, person-related bullying, and organizational injustice) serving as observed variables. The study treated cognitive reappraisal, expressive suppression, and work engagement as endogenous observed variables, and adaptive performance as an endogenous latent variable, with its two dimensions (handling emergencies and unpredictable situations, demonstrating culture and interpersonal adaptability) serving as observed variables. Additionally, the study incorporated marital status, education level, years of work experience, daily working hours, and history of occupational exposure as control variables. Path coefficient tests revealed that these control variables had minimal path coefficients with adaptive performance and contributed negligibly to the overall model. Therefore, following the principle of model parsimony in structural equation modeling, the study retained only the main influence paths among workplace bullying, cognitive reappraisal, expressive suppression, work engagement, and adaptive performance in the final model to construct a more parsimonious and effective model. The standardized path coefficients are presented in Figure 1.

The indexes for model fit were as follows:*χ^2^*/*df* = 4.709, CFI = 0.988, TLI = 0.976, SRMR = 0.026, RMSEA = 0.061. The current study adopted a large sample size, which may affect the value of *χ^2^/df* and RMSEA (39). In general, the fitting index of the model is acceptable.

All path coefficients in the model were significant (Table 3). Specifically:

**Table 3:** Results of structural model testing.

Path	Std (*β*)	S. E.	C. R.	*P*
WPB → Cognitive reappraisal	−0.212	0.036	−5.901	<0.001
WPB → Expressive suppression	0.144	0.032	4.509	<0.001
WPB → Work engagement	−0.168	0.033	−5.137	<0.001
WPB → Adaptive performance	−0.175	0.037	−4.737	<0.001
Cognitive reappraisal → Work engagement	0.484	0.037	12.976	<0.001
Cognitive reappraisal → Adaptive performance	0.355	0.045	7.953	<0.001
Expressive suppression → Work engagement	−0.108	0.033	−3.266	0.001
Expressive suppression → Adaptive performance	−0.067	0.030	−2.243	0.025
Work engagement → Adaptive performance	0.360	0.045	7.992	<0.001

WPB negatively predicted cognitive reappraisal (*β* = −0.212, *p* < 0.001), work engagement (*β* = −0.168, *p* < 0.001), and adaptive performance (*β* = −0.175, *p* < 0.001), while positively predicting expressive suppression (*β* = 0.144, *p* < 0.001).

Cognitive reappraisal positively predicted work engagement (*β* = 0.484, *p* < 0.001) and adaptive performance (*β* = 0.355, *p* < 0.001).

Expressive suppression negatively predicted work engagement (*β* = −0.108, *p* = 0.001) and adaptive performance (*β* = −0.067, *p* = 0.025).

Work engagement positively predicted adaptive performance (*β* = 0.360, *p* < 0.001).

The bias-corrected bootstrap method with 5,000 samples was used to estimate the 95% confidence interval of the indirect effect parameter. As demonstrated in Table 4, the model revealed statistically significant mediating effects across all pathways, which is indicated by the 95% BC CI (Bias-Corrected Confidence Interval) excluding zero. WPB exerted a significant direct negative effect on adaptive performance (*β* = −0.175, 95% BC CI [−0.247, −0.106]), supporting Hypothesis 1. Additionally, WPB reduced adaptive performance through three significant single mediation paths: cognitive reappraisal (*β* = −0.075, 95% BC CI [−0.114, −0.047]), expressive suppression (*β* = −0.010, 95% BC CI [−0.022, −0.002]), and work engagement (*β* = −0.060, 95% BC CI [−0.091, −0.035]), supporting Hypotheses 2a, 2b, and 2c. Furthermore, two chain mediation pathways were significant: the sequential path through cognitive reappraisal → work engagement (*β* = −0.037, 95% BC CI [−0.054, −0.024]) and the path through expressive suppression → work engagement (*β* = −0.006, 95% BC CI [−0.012, −0.002]), supporting Hypothesis 3. The total indirect effect was significant (*β* = −0.188, 95% BC CI [−0.238, −0.138]), accounting for 51.8% of the total effect.

**Table 4:** The mediating effect of emotion regulation and work engagement in the association between WPB and adaptive performance.

Path	Std (*β*)	S. E.	LLCI	ULCI
Total effect	−0.363	0.047	−0.447	−0.265
Direct effect	−0.175	0.037	−0.247	−0.106
Indirect effect	−0.188	0.025	−0.238	−0.138
WPB → Cognitive reappraisal → Adaptive performance	−0.075	0.017	−0.114	−0.047
WPB → Expressive suppression → Adaptive performance	−0.010	0.005	−0.022	−0.002
WPB → Work engagement → Adaptive performance	−0.060	0.014	−0.091	−0.035
WPB → Cognitive reappraisal → Work engagement → Adaptive performance	−0.037	0.007	−0.054	−0.024
WPB → Expressive suppression → Work engagement → Adaptive performance	−0.006	0.002	−0.012	−0.002

## 4 Discussion

Based on JD-R and Emotion regulation theories, this study explores how workplace bullying (negative job demands) affects adaptive performance through Emotion regulation strategies (personal resources) and work engagement. The results indicate that workplace bullying not only directly negatively predicts adaptive performance but also indirectly influences it through cognitive reappraisal, expressive suppression, and work engagement, with a total mediating effect of 51.8 %.

### 4.1 Effect of workplace bullying on adaptive performance

The present study found a significant negative correlation between workplace bullying and adaptive performance among junior nurses, aligning with previous research (19). According to the Job Demands-Resources (JD-R) theory, nurses who experience bullying not only face high job demands such as overtime work, increased task load, or unfair treatment, but also suffer from a dual deficiency in perceived psychological safety and organizational support due to the threatening work environment (23). When both personal and organizational resources are insufficient to cope with continuously escalating job demands, their adaptive behaviors necessary for handling complex problems inevitably decrease, ultimately severely impairing adaptive performance (40). It is worth noting that the effects of different types of bullying on performance may differ. Pei et al. (41) found in a longitudinal study of nurses in Shandong that psychological violence had a significant negative impact on job performance, while the effects of physical violence were not substantial. This finding provides a reference for the present study. Future research could employ multivariate path analysis or Bayesian estimation methods to conduct in-depth comparisons of the specific mechanisms through which different types of bullying affect adaptive performance, thereby providing more precise empirical evidence for targeted intervention measures.

### 4.2 Mediation through cognitive reappraisal and work engagement

The present study found that workplace bullying not only directly affects adaptive performance but also exerts an indirect influence via a chain-mediated path involving cognitive reappraisal and work engagement. Workplace bullying was negatively correlated with cognitive reappraisal, consistent with previous findings that healthcare workers repeatedly exposed to adverse work environments tend to rely more on negative coping mechanisms rather than actively engaging in cognitive reappraisal (42). When individuals face chronic exposure to a bullying environment, they must allocate their limited cognitive resources to coping with threats and processing negative emotions, leaving fewer resources for constructive cognitive restructuring (43). This resource depletion makes it difficult for nurses to muster enough psychological energy to transform negative experiences into manageable challenges. Nevertheless, for nurses who can maintain some capacity for cognitive reappraisal despite resource depletion, this strategy helps them recover psychological resources, activate neural mechanisms associated with cognitive control, and flexibly adjust cognitive patterns in challenging situations (25). Previous studies have confirmed that cognitive reappraisal can promote employees’ work engagement (29), enabling them to tackle work challenges with greater enthusiasm and focus, which contributes to enhanced adaptive performance.

### 4.3 Mediation through expressive suppression and work engagement

This study further identified that expressive suppression and work engagement constitute another chain mediation pathway through which workplace bullying influences adaptive performance. As a high-risk group for bullying, some junior nurses may choose to remain silent to maintain interpersonal “harmony” or due to power pressure (44), internalizing the negative emotions stemming from bullying and suppressing their emotional experiences to alleviate distress. This coping style objectively increases their use of expressive suppression. Consistent with previous research, repeated exposure to uncontrollable and demeaning work environments diminishes individuals’ perceived efficacy in modifying their emotional experiences, thereby impairing their emotional regulation capacity (42). Under this coping pattern, sustained expressive suppression not only exacerbates individuals’ psychological distress and emotional disturbance (26), but also continuously consumes limited cognitive resources to maintain the suppression of emotional expression. This resource depletion subsequently undermines their ability to effectively mobilize internal and external resources to adapt to stressful situations (45). This resource loss, resulting from the combination of emotional internalization and cognitive monitoring, is associated with reduced work engagement, leaving nurses feeling exhausted and detached at work. Previous studies have confirmed that low work engagement further weakens an individual’s ability to cope with environmental changes, which ultimately hinders the development of adaptive performance (46).

The above findings are consistent with both the Emotion Regulation Theory and the JD-R theory. The Emotion Regulation Theory elucidates the distinct mechanisms of cognitive reappraisal and expressive suppression: the former helps individuals maintain psychological balance by altering their cognition, while the latter, when used repeatedly, depletes cognitive resources and leads to a rebound of accumulated emotions. The JD-R theory further explains how these two strategies, as personal resources, influence work outcomes: cognitive reappraisal activates a gain pathway to enhance work engagement, whereas expressive suppression triggers a loss pathway, resulting in decreased work engagement. Work engagement, as a key motivational state, ultimately transmits the effects of emotion regulation to adaptive performance.

It is worth noting that in this study, expressive suppression had a significant adverse predictive effect on work engagement (*β* = −0.108) and indirectly influenced adaptive performance through work engagement (indirect effect *β* = −0.006). However, its effect size was substantially smaller than that of the gain pathway through cognitive reappraisal (*β* = −0.037), with a confidence interval approaching zero. This finding differs to some extent from previous research: for instance, Zeng et al. (47) found no significant direct effect of expressive suppression on work engagement, Wobeto et al. (48) even observed a positive predictive relationship, while Huang et al. (28) reported a significant adverse impact (*β* = −0.23). Such inconsistencies suggest that different contextual and cultural factors may moderate the role of expressive suppression. The relatively small effect size in this study indicates that the adverse effects of expressive suppression may be partially buffered among junior nurses. Jones et al. (49) found that the emotion-regulatory effects of expressive suppression are context-dependent. Under extreme stress situations, it may serve a short-term protective function by temporarily blocking emotional expression and helping individuals focus cognitive resources on urgent tasks (50). Therefore, in the context of workplace bullying, the negative effects of resource depletion, although present, are attenuated due to the specific nature of the situation, thereby exhibiting a partial buffering effect.

Furthermore, the role of expressive suppression is also culture-specific: East Asian cultures emphasize emotional control and interpersonal harmony, where moderately suppressing negative emotions may help avoid conflict and maintain team harmony (51, 52). Such cultural norms may partially offset the psychological resource depletion associated with expressive suppression, leading to an overall effect size smaller than that observed in Western studies. In summary, the role of expressive suppression is not a singularly adverse effect but rather a complex outcome shaped by the interplay of contextual urgency and cultural norms, which provides a reasonable explanation for its relatively small effect size observed in this study.

## 5 Strengths and limitations

This study has several strengths. First, by integrating Emotion Regulation Theory with the Job Demands-Resources (JD-R) Theory, it constructed and tested a dual-pathway “gain-loss” chain mediation model of workplace bullying’s impact on adaptive performance. The findings not only revealed the differentiated mechanisms through which cognitive reappraisal and expressive suppression transmit to performance via work engagement but also introduced a culture-specific perspective, proposing that East Asian cultural norms may buffer the negative effects of expressive suppression—providing a novel interpretative framework for resolving cross-cultural inconsistencies regarding this strategy. Second, by focusing on junior nurses as a distinct vulnerable population, this study clarified the internal pathways through which adaptive performance is compromised following bullying exposure, offering empirical evidence to inform targeted interventions in healthcare settings.

However, several limitations should be acknowledged. First, the cross-sectional design precludes causal inferences and fails to capture the dynamic evolution of adaptive performance over time. Second, although Harman’s test and the ULMC approach indicated no serious common method bias, these methods have inherent insensitivity, and self-reported, single-source data may still introduce bias. Third, monetary incentives and the sensitive nature of workplace bullying may have induced response biases (e.g., careless responding, social desirability). Fourth, convenience sampling from 17 hospitals in Southwest China (a single cultural context) limits generalizability nationwide and cross-culturally. Fifth, assessing workplace bullying as a unidimensional construct may obscure differential effects of personal, work-related, and physical bullying on adaptive performance. Future studies should adopt longitudinal or multi-source designs (e.g., supervisor-rated performance), employ non-monetary incentives, and incorporate implicit measures or peer reports to capture sensitive constructs more accurately. Multicenter studies using probability sampling across diverse geographic and cultural contexts are needed to enhance representativeness. Additionally, research should also disaggregate bullying subtypes, examine cultural moderators, and explore other mediators (e.g., psychological capital) and boundary conditions (e.g., leadership style) to clarify when and for whom workplace bullying effects on adaptive performance are amplified or buffered.

## 6 Conclusion

Junior nurses’ adaptive performance plays a crucial role in their career development, team effectiveness, and patient care quality. Therefore, prioritizing effective interventions to enhance it is essential. Workplace bullying significantly negatively affects the adaptive performance. Additionally, Emotion regulation and work engagement mediate the relationship between workplace bullying and adaptive performance. Interventions should reduce workplace bullying and address methods of emotional expressive suppression, promoting cognitive reappraisal and job engagement. These strategies can directly or indirectly enhance the adaptability and performance of junior nurses.

## 7 Implications for nursing management

The chain mediation model offers three evidence-informed directions for enhancing junior nurses’ adaptive performance. First, given bullying’s direct negative effects, organizations should prioritize early identification and reduction of bullying behaviors through fostering respectful work environments. Second, the significant mediation pathways highlight emotion regulation as a critical intervention point. By helping nurses cognitively reframe bullying incidents, they better protect their psychological resources and maintain focus. Furthermore, given the culture-specific differences in expressive suppression observed in this study, managers should not impose a single emotion regulation strategy. Instead, they should support nurses in flexibly adapting their regulatory approaches to different clinical situations, thereby alleviating the cognitive resource depletion associated with rigid emotion regulation. Third, work engagement emerged as a critical transmission mechanism. Thus, sustaining nurses’ vigor and dedication through constructive feedback, meaningful recognition, and professional guidance is warranted. By addressing these interconnected factors: reducing exposure to bullying, supporting adaptive emotion regulation, and fostering work engagement, healthcare institutions can more effectively enhance the adaptive performance and stability of their junior nursing workforce.

## Data Availability

The raw data supporting the conclusions of this article will be made available by the authors, without undue reservation.
