# Supplementary material for: The relationship between workplace bullying and adaptive performance in junior nurses: the mediating role of emotion regulation and work engagement
Source: Front Public Health. 2026 Mar 25;14:1805252. doi: 10.3389/fpubh.2026.1805252 (PMC13057310; doi:10.3389/fpubh.2026.1805252)
Supplement: Supplementary file 1 [file Table_1.docx]

# A Study on the Current Status and Influencing Factors of Adaptive performance among Junior Nurses

Dear Nursing Colleagues:

Hello, we sincerely invite you to participate in a research survey (Ethics Approval No.: 2024ER112-1). Please answer according to your actual situation; there are no right or wrong answers. This survey consists of 2 parts: ① Informed Consent Form; ② Questionnaire. It will take approximately 8-12 minutes to complete. To thank you for your support, you will have the opportunity to draw a red packet after submitting this questionnaire. Before you decide whether to participate, please read the following informed consent form carefully. If you have any questions, please contact Siyuan Li. (Email: lsy33801@163.com)

# Informed Consent Form

Project Title: A Study on the Current Status and Influencing Factors of Adaptive performance among Junior Nurses

Principal Investigators: Xuemei Wei (Chief Nursing Officer), Siyuan Li, Yuze Wu (Master's Degree) (Affiliated Hospital of North Sichuan Medical College), and Lanjun Luo (PhD) (School of Management, North Sichuan Medical College), etc.

Research Purpose: To understand the current status of adaptive performance among junior nurses and its influencing factors, explore the mechanisms of these factors, and provide evidence for nursing managers to develop targeted intervention strategies.

Research Process: Collect relevant research information through an online questionnaire survey.

Confidentiality of Personal Information and Records: Your survey information will be anonymous. The researchers will keep it strictly confidential, and all questionnaire data will be destroyed centrally after the study concludes.

You have the right to voluntarily choose to participate in the survey or withdraw midway without any negative consequences.

Contact Person : Siyuan Li, Email: lsy33801@163.com

I have read the above introduction to this survey and understand the potential risks involved. I ______ to participate in this survey.[Singlechoice] *

| ○Agree |
| --- |
| ○Disagree |

**Demographic Information**

1. Gender [Singlechoice] *

○ Male

○ Female

2. Marital status [Singlechoice] *

○ Unmarried

○ Married

3. Highest level of nursing education [Singlechoice] *

○ Technical secondary school

○ Associate degree (junior college)

○ Bachelor's degree

○ Master's degree or above

4. Hospital grade [Singlechoice] *

○ Grade III A

○Grade III B

○ Grade II A

○ Grade II B

5. Years of clinical nursing experience (excluding internship) [Singlechoice] *

○ < 6 months

○ 6 months – 1 year

○ >1 – 3 years

○ >3 – 5 years

6. Average work hours per day [Singlechoice] *

○ ≤ 8 hours

○ > 8 hours

7. Average monthly personal income (RMB) [Singlechoice] *

○ < 3000

○ 3000 – 6000

○ 6001 – 9000

○ > 9000

8. History of occupational exposure (e.g., needlestick injury, radiation exposure) [Singlechoice] *

○ Yes

○ No

**Workplace bullying refers to adverse, systematic, repeated, and persistent negative behaviors such as offense, humiliation, harassment, and exclusion directed at an employee by one or more individuals in the workplace.**

Please select the most appropriate option based on your experiences at work during the last 6 months.*

|  | Never | Now and then | Monthly | Weekly | Daily |
| --- | --- | --- | --- | --- | --- |
| Someone withholding information which affects your performance | ○ | ○ | ○ | ○ | ○ |
| Being ordered to do work below your level of competence | ○ | ○ | ○ | ○ | ○ |
| Having your opinions ignored | ○ | ○ | ○ | ○ | ○ |
| Being given tasks with unreasonable deadlines | ○ | ○ | ○ | ○ | ○ |
| Excessive monitoring of your work | ○ | ○ | ○ | ○ | ○ |
| Pressure not to claim something to which by right you are entitled (e.g. sick leave, holiday entitlement, travel expenses) | ○ | ○ | ○ | ○ | ○ |
| Being exposed to an unmanageable workload | ○ | ○ | ○ | ○ | ○ |
| Being humiliated or ridiculed in connection with your work | ○ | ○ | ○ | ○ | ○ |
| Having key areas of responsibility removed or replaced with more trivial or unpleasant tasks | ○ | ○ | ○ | ○ | ○ |
| Spreading of gossip and rumours about you | ○ | ○ | ○ | ○ | ○ |
| Being ignored or excluded | ○ | ○ | ○ | ○ | ○ |
| Having insulting or offensive remarks made about your person, attitudes or your private life | ○ | ○ | ○ | ○ | ○ |
| Hints or signals from others that you should quit your job | ○ | ○ | ○ | ○ | ○ |
| Repeated reminders of your errors or mistakes | ○ | ○ | ○ | ○ | ○ |
| Being ignored or facing a hostile reaction when you approach | ○ | ○ | ○ | ○ | ○ |
| Persistent criticism of your errors or mistakes | ○ | ○ | ○ | ○ | ○ |
| Practical jokes carried out by people you don't get along with | ○ | ○ | ○ | ○ | ○ |
| Having allegations made against you | ○ | ○ | ○ | ○ | ○ |
| Being the subject of excessive teasing and sarcasm | ○ | ○ | ○ | ○ | ○ |
| Being shouted at or being the target of spontaneous anger | ○ | ○ | ○ | ○ | ○ |
| Intimidating behaviours such as finger-pointing, invasion of personal space, shoving, blocking your way | ○ | ○ | ○ | ○ | ○ |
| Threats of violence or physical abuse or actual abuse | ○ | ○ | ○ | ○ | ○ |

**Adaptive Performance**

Please rate the extent to which you agree with the following statements.

1.Controlling my emotions when dealing with urgent problems[Singlechoice] *

| Very Dissatisfied | ○1 | ○2 | ○3 | ○4 | ○5 | Very Satisfied |
| --- | --- | --- | --- | --- | --- | --- |

2.Taking step-by-step measures to solve urgent problems[Singlechoice] *

| Very Dissatisfied | ○1 | ○2 | ○3 | ○4 | ○5 | Very Satisfied |
| --- | --- | --- | --- | --- | --- | --- |

3.Remaining calm when work pressure is high[Singlechoice] *

| Very Dissatisfied | ○1 | ○2 | ○3 | ○4 | ○5 | Very Satisfied |
| --- | --- | --- | --- | --- | --- | --- |

4.Thinking clearly and prioritizing well when handling urgent problems[Singlechoice] *

| Very Dissatisfied | ○1 | ○2 | ○3 | ○4 | ○5 | Very Satisfied |
| --- | --- | --- | --- | --- | --- | --- |

5.Remaining calm when work schedules become very stressful[Singlechoice] *

| Very Dissatisfied | ○1 | ○2 | ○3 | ○4 | ○5 | Very Satisfied |
| --- | --- | --- | --- | --- | --- | --- |

6.Handling urgent problems objectively[Singlechoice] *

| Very Dissatisfied | ○1 | ○2 | ○3 | ○4 | ○5 | Very Satisfied |
| --- | --- | --- | --- | --- | --- | --- |

7.Coming up with several alternative solutions for dealing with urgent problems[Singlechoice] *

| Very Dissatisfied | ○1 | ○2 | ○3 | ○4 | ○5 | Very Satisfied |
| --- | --- | --- | --- | --- | --- | --- |

8.Integrating into other different values, customs, and cultures[Singlechoice] *

| Very Dissatisfied | ○1 | ○2 | ○3 | ○4 | ○5 | Very Satisfied |
| --- | --- | --- | --- | --- | --- | --- |

9.Modifying my behavior to adapt to other cultures, customs, and habits[Singlechoice] *

| Very Dissatisfied | ○1 | ○2 | ○3 | ○4 | ○5 | Very Satisfied |
| --- | --- | --- | --- | --- | --- | --- |

10.Working well with people who have different personalities[Singlechoice] *

| Very Dissatisfied | ○1 | ○2 | ○3 | ○4 | ○5 | Very Satisfied |
| --- | --- | --- | --- | --- | --- | --- |

11.Understanding the organizational climate, development direction, and values of the hospital[Singlechoice] *

| Very Dissatisfied | ○1 | ○2 | ○3 | ○4 | ○5 | Very Satisfied |
| --- | --- | --- | --- | --- | --- | --- |

12.Actively learning about the work atmosphere, needs, and values of other departments[Singlechoice] *

| Very Dissatisfied | ○1 | ○2 | ○3 | ○4 | ○5 | Very Satisfied |
| --- | --- | --- | --- | --- | --- | --- |

13.Adjusting my behavior when necessary[Singlechoice] *

| Very Dissatisfied | ○1 | ○2 | ○3 | ○4 | ○5 | Very Satisfied |
| --- | --- | --- | --- | --- | --- | --- |

14.Understanding the meaning of behaviors in other cultures and adjusting my actions accordingly[Singlechoice] *

| Very Dissatisfied | ○1 | ○2 | ○3 | ○4 | ○5 | Very Satisfied |
| --- | --- | --- | --- | --- | --- | --- |

15.Maintaining good relationships with people from different cultural backgrounds[Singlechoice] *

| Very Dissatisfied | ○1 | ○2 | ○3 | ○4 | ○5 | Very Satisfied |
| --- | --- | --- | --- | --- | --- | --- |

**Emotion Regulation Questionnaire (ERQ)**

Please indicate the extent to which you agree with the following statements.

1. I control my emotions by changing the way I think about the situation I'm in.[Singlechoice] *

| Strongly Disagree | ○1 | ○2 | ○3 | ○4 | ○5 | ○6 | ○7 | Strongly Agree |
| --- | --- | --- | --- | --- | --- | --- | --- | --- |

1. When I want to feel less negative emotion, I change the way I'm thinking about the situation.[Singlechoice] *

| Strongly Disagree | ○1 | ○2 | ○3 | ○4 | ○5 | ○6 | ○7 | Strongly Agree |
| --- | --- | --- | --- | --- | --- | --- | --- | --- |

1. When I want to feel more positive emotion, I change the way I'm thinking about the situation.[Singlechoice] *

| Strongly Disagree | ○1 | ○2 | ○3 | ○4 | ○5 | ○6 | ○7 | Strongly Agree |
| --- | --- | --- | --- | --- | --- | --- | --- | --- |

1. When I want to feel more positive emotion (such as joy or amusement), I change what I'm thinking about.[Singlechoice] *

| Strongly Disagree | ○1 | ○2 | ○3 | ○4 | ○5 | ○6 | ○7 | Strongly Agree |
| --- | --- | --- | --- | --- | --- | --- | --- | --- |

1. When I want to feel less negative emotion (such as sadness or anger), I change what I'm thinking about.[Singlechoice] *

| Strongly Disagree | ○1 | ○2 | ○3 | ○4 | ○5 | ○6 | ○7 | Strongly Agree |
| --- | --- | --- | --- | --- | --- | --- | --- | --- |

1. When I'm faced with a stressful situation, I make myself think about it in a way that helps me stay calm.[Singlechoice] *

| Strongly Disagree | ○1 | ○2 | ○3 | ○4 | ○5 | ○6 | ○7 | Strongly Agree |
| --- | --- | --- | --- | --- | --- | --- | --- | --- |

1. I keep my emotions to myself.[Singlechoice] *

| Strongly Disagree | ○1 | ○2 | ○3 | ○4 | ○5 | ○6 | ○7 | Strongly Agree |
| --- | --- | --- | --- | --- | --- | --- | --- | --- |

1. I control my emotions by not expressing them.[Singlechoice] *

| Strongly Disagree | ○1 | ○2 | ○3 | ○4 | ○5 | ○6 | ○7 | Strongly Agree |
| --- | --- | --- | --- | --- | --- | --- | --- | --- |

1. When I am feeling negative emotions, I make sure not to express them.[Singlechoice] *

| Strongly Disagree | ○1 | ○2 | ○3 | ○4 | ○5 | ○6 | ○7 | Strongly Agree |
| --- | --- | --- | --- | --- | --- | --- | --- | --- |

1. When I am feeling positive emotions, I am careful not to express them.[Singlechoice] *

| Strongly Disagree | ○1 | ○2 | ○3 | ○4 | ○5 | ○6 | ○7 | Strongly Agree |
| --- | --- | --- | --- | --- | --- | --- | --- | --- |

**Work Engagement Questionnaire**

Please rate the extent to which you agree with the following statements.

1. At my work, I feel bursting with energy[Singlechoice] *

| Very Dissatisfied | ○1 | ○2 | ○3 | ○4 | ○5 | Very Satisfied |
| --- | --- | --- | --- | --- | --- | --- |

1. I am enthusiastic about my job[Singlechoice] *

| Very Dissatisfied | ○1 | ○2 | ○3 | ○4 | ○5 | Very Satisfied |
| --- | --- | --- | --- | --- | --- | --- |

1. I am immersed in my work.[Singlechoice] *

| Very Dissatisfied | ○1 | ○2 | ○3 | ○4 | ○5 | Very Satisfied |
| --- | --- | --- | --- | --- | --- | --- |
